# Younger age and induction failure predict outcomes in infant leukemia: 30 years of experience in a tertiary center

**DOI:** 10.3389/fped.2023.1166176

**Published:** 2023-05-30

**Authors:** Bárbara Ochoa-Fernández, Víctor Galán-Gómez, Pilar Guerra-García, Sonsoles Sanromán, Isabel Martínez, David Bueno, Yasmina Mozo, Luisa Sisinni, Itsaso Losantos, Berta González, Antonio Pérez-Martínez

**Affiliations:** ^1^ Pediatric Haemato-Oncology Department, La Paz University Hospital, Madrid, Spain; ^2^ Translational Research in Pediatric Oncology, Hematopoietic Transplantation and Cell Therapy, Hospital La Paz Institute for Health Research—IdiPAZ, Madrid, Spain

**Keywords:** infant leukemia, acute lymphoblastic leukemia, acute myeloid leukemia, *KMT2A* rearrangement, hematopoietic stem cell transplantation, infant leukemia

## Abstract

**Objectives:**

This study aimed to evaluate the characteristics and outcomes of infant patients with leukemia.

**Methods:**

A retrospective analysis was conducted in a cohort of 39 patients diagnosed with infant leukemia from 1990 to 2020 who underwent treatment at the pediatric hemato-oncology department of a tertiary hospital in Madrid, Spain.

**Results:**

Of the 588 diagnosed cases of childhood leukemia, 39 (6.6%) cases were infant leukemia. The 5-year event-free survival and the 5-year overall survival were 43.6% (SE 4.1) and 46.5% (SD 24.08), respectively. In a univariate analysis, a younger age at diagnosis was associated with poorer outcomes (*p* = 0.027), as was induction failure (*p* = 0.0024). Patients treated with hematopoietic stem cell transplantation had better outcomes than non-transplanted patients (*p* = 0.001); however, the group comparisons that exclude patients who were unable to undergo transplantation due to refractoriness/relapse or death during treatment showed no significant differences.

**Conclusions:**

The main risk factors that affected survival in our study were an age younger than 6 months and a poor response to induction therapy. It is important to identify poor prognostic factors in this population in order to seek different approaches that could improve outcomes.

## Introduction

Acute leukemia is the most common malignancy diagnosed in children. Although survival rates have greatly increased over time, infant leukemia (IL) remains a challenge, and therapeutic improvements are needed.

The term “infant leukemia” refers to acute leukemia diagnosed before the first year of life and represents a distinctive subgroup of patients with particular clinical and biological characteristics. Infant leukemia is a rare disease, with an estimated incidence of 45 cases per million in the United States, equivalent to 175 cases per year (1, 2). During the recent decades, infant leukemia comprised 5.5% of the cases of childhood leukemia in Spain, amounting to 186 cases (3). Within the infant group, leukemia is the second most common cancer after neuroblastoma and accounts for 16.4% of infant malignancies (4).

The particularities of infant leukemia include a female predominance (in contrast to male predominance in older children) and an increased incidence rate of acute myeloblastic leukemia (AML), a rate approximately twice that for older children, in whom there is a clear predominance of acute lymphoblastic leukemia (ALL). Clinically, the onset of infant leukemia tends to present with more aggressive features, including higher white blood cell (WBC) counts, hepatosplenomegaly, involvement of the central nervous system (CNS), and skin infiltration (5).

In terms of genetics, *KMT2A* or the lysine methyltransferase 2A gene (previously known as mixed-lineage leukemia, *MLL*) rearrangements are a primary characteristic of infant leukemia. *KMT2A* rearrangements (*KMT2A*-r) are balanced chromosomal translocations that appear in 5% of cases of childhood leukemia (80% of infant ALL cases and 50% of infant AML cases) (2). *KMT2*-r in ALL is associated with CD10 negativity and co-expression of myeloid antigens. *KMT2A*-r also confers different prognostic connotations between infant ALL and AML. The presence of *KMT2A*-r in ALL is associated with poor outcomes (6).

Although the long-term event-free survival (EFS) rates for childhood leukemia are near 80%, the prognosis for infants diagnosed with leukemia is unfavorable, and the treatment of this high-risk patient group remains a major challenge despite the use of intensified standard therapies (4, 6). The indication for hematopoietic stem cell transplantation (HSCT) is restricted to specific subgroups with risk factors of poor prognosis and is controversial in terms of efficacy and potential for acute and late toxicity (7).

The objective of this retrospective descriptive study is to reflect the experience of a single tertiary center over the past three decades.

## Materials and methods

We retrospectively analyzed a cohort of 39 patients diagnosed with infant leukemia from 1990 to 2020, who underwent treatment at the pediatric hemato-oncology department of a tertiary hospital in Madrid, Spain.

Infant leukemia was defined as acute leukemia diagnosed during the first year of life. The leukemia diagnosis was based on cytomorphology, immunophenotyping, cytogenetics (karyotype and fluorescence *in situ* hybridization, FISH), and molecular biology of bone marrow aspirate.

The variables that were analyzed included patients’ characteristics, such as age, sex, initial signs and symptoms, laboratory findings at diagnosis, CNS involvement, immunophenotype, HSCT, relapse, and follow-up (Table 1). Data were collected from the medical records of the hospital.

**Table 1:** Summary of the patients’ characteristics.

Infant leukemia	Total
Patients, *n*	39
Sex, *n* (%)	
Male	15 (38.5)
Female	24 (61.5)
Age (months), mean (SD)	5.59 (3.5)
Diagnosis, *n* (%)	
ALL	27 (69)
	Pro-B: 13 (33)
	Pre-B: 9 (23)
	Common B: 4 (10)
	Mature T cell: 1 (3)
AML	
	11 (28)
	M0: 1 (3)
	M2: 1 (3)
	M4: 1 (3)
	M5: 3 (8)
	M7: 5 (13)
MPAL	1 (3)
*KMT2A*-r, *n* (%)	22 (59.5)
Translocation *KMT2A*-r, *n*	
	t(4;11): 10
	t(9;11): 3
	t(11;19): 3
	t(10;11): 3
	Other: 3
CD10 (CALLA), *n* (%)	
Negative	28 (72)
WBCs at diagnosis, cells/mm^3^	
Median (IQR)	100.000 (297.000)
WBC > 300,000/mm^3^, *n* (%)	12 (31)
Hepatosplenomegaly, *n* (%)	28 (71)
Skin infiltration, *n* (%)	7 (18)
CNS involvement, *n* (%)	2 (5)
Induction failure, *n* (%)	6 (15.3)
Relapse, *n* (%)	14 (36)
HSCT, *n* (%)	26 (66.6)
Autologous:	4 (15.3)
Allogeneic:	22 (84.6)
Status, *n* (%)	
Alive	20 (51)
5-year EFS, % (SE)	43.6% (4.1)
5-year OS, % (SE)	46.5% (24.08)

ALL, acute lymphoblastic leukemia; AML, acute myeloid leukemia; CNS, central nervous system; EFS, event-free survival; HSCT, hematopoietic stem cell transplantation; IQR, interquartile range; MPAL, mixed-phenotype acute leukemia; OS, overall survival; SE, standard error; TRM, transplant-related mortality; WBC, white blood cell.

Patients with ALL were initially treated within the protocol from the Spanish Cooperative Group SHOP/ALL (1990–2016) and according to the INTERFANT-06 protocol since 2018 (8, 9). Patients with AML were treated within the SHOP/AML protocols at the beginning of the cohort study (1990–2014) and within the NOPHO-DBH AML 2012 trial since 2018.

This retrospective data collection study was approved by the ethics committee of University Hospital La Paz (Code PI-4575), and written informed consent to participate in this study was obtained from the parents/legal guardians.

### Statistical analysis

Qualitative data are presented with absolute and relative frequencies. Quantitative data are presented as mean ± standard deviation (SD) if they followed normality and as median and interquartile ranges if not. Normality was studied employing the Kolmogorov–Smirnov test. The association between qualitative variables was analyzed with the *χ*^2^ test or Fisher's exact test.

Survival was determined by the 5-year overall survival (OS) rate, employing a Kaplan–Meier analysis. EFS was defined as the time from diagnosis to relapse or death from any cause. Transplant-related mortality (TRM) was defined as death after HSCT without disease progression or relapse. We performed a survival analysis employing a Kaplan–Meier estimator (log-rank tests were employed to compare the survival functions by groups) and Cox regression (to obtain the risks associated with the significant variables).

Differences were considered statistically significant at *p* ≤ 0.05, with a 95% confidence interval. The statistical analysis was conducted with the SAS 9.3 software (SAS Institute, Cary, NC, United States).

## Results

A total of 588 patients were diagnosed with childhood leukemia during this period. Of these, 39 (6.6%) were diagnosed with infant leukemia (patients’ demographics are summarized in Table 1). Due to the long period of the study, we divided it into two groups: an early period (from 1990 to 2005) and a late period (from 2006 to 2020) (Table 2).

**Table 2:** Evolution of characteristics over the years.

Period	1990–2005			2006–2020		
Diagnosis	ALL	AML	Total	ALL	AML	Total
	10	3	13	17	8	25
Age <6 months, *n* (%)	6 (60)	1 (33.3)	7	9 (52.9)	4 (50)	13
*KMT2A*-r, *n* (%)	6 (60)	0	6	14 (82.3)	2 (25)	17
Induction failure, *n* (%)	1 (10)	2 (66.6)	3	2 (11.7)	0	2
Relapse, *n* (%)	3 (30)	1 (33.3)	4	8 (47)	1 (12.5)	9
HSCT, *n* (%)	5 (50)	2 (66.6)	7	13 (76.4)	5 (62.5)	18
Type HSCT						
Autologous	2	0	2	1	1	2
Identical	1	2	3	8	3	11
Haploidentical	2	0	2	4	2	6
Status alive, *n* (%)	3 (30)	2 (66.6)	5	9 (52.9)	6(75)	15

ALL, acute lymphoblastic leukemia; AML, acute myeloid leukemia; HSCT, hematopoietic stem cell transplantation.

There were 24 (61.5%) girls and 15 (38.5%) boys, with a mean age at diagnosis of 5.5 months (SD 3.5). A total of 27 (69%) patients were diagnosed with ALL, 11 (28%) with AML, and 1 patient with mixed-phenotype acute leukemia. At diagnosis, 12 (30.8%) patients had hyperleukocytosis >300,000 cells/mm^3^, 22 (59.5%) harbored *KMT2A*-r, and 2 (5.1%) had CNS involvement. Induction failure and relapse occurred in 6 (15.3%) and 14 (35.9%) patients, respectively. The median duration of the first remission before relapse was 4 (3.1–8.4) months.

A total of 26 (66.6%) patients underwent HSCT (Supplementary Table 1), 24 (92.3%) after achieving the first complete remission, 4 (15.4%) of them with autologous SCT. With a median follow-up of 15 months, the 5-year EFS and 5-year OS were 43.6% (SE 4.1) and 46.5% (SE 24.08), respectively. In a univariate analysis, a younger age at diagnosis was associated with poorer outcomes (*p* = 0.027), as was the failure to achieve remission after induction therapy (*p* = 0.0024) (Table 3). In our series, the patients who underwent HSCT had better outcomes than the non-transplanted patients (*p* = 0.001); however, group comparison taking into account the criteria for not performing HSCT did not show differences (see Table 4). TRM was 31.6%, and relapse after HSCT occurred in 7 (17.9%) patients.

**Table 3:** Univariate analysis of prognostic factors.

	5-year OS (%)	HR (95% CI)	*p*-value
AML	71.6		0.131
ALL	38.4		
*KMT2A*-r			0.985
Yes	45.3		
No	46.3		
Induction failure			0.024
No	52.4	0.32	
Yes	16.7	(0.11-.0.91)	
HSCT			0.001
Yes	60.4	0.18	
No	18.5	(0.07–0.46)	
WBC			0.464
>300,000	37.5		
<300,000	51.2		
AGE			0.027
<6 months	36.5	0.85	
>6 months	60.5	(0.73–0.98)	

ALL, acute lymphoblastic leukemia; AML, acute myeloid leukemia; EFS, event-free survival; HSCT, hematopoietic stem cell transplantation; HR, hazard ratio; OS, overall survival; WBC, white blood count.

**Table 4:** Comparison between transplanted and non-transplanted patients.

	No HSCT	HSCT	*p-value*
No. of patients, *n* (%)	13 (33.3)	26 (66.6)	
Sex, *n* (%)			0.036
Male	8 (61.5)	7 (26.9)	
Female	5 (38.5)	19 (73.1)	
Age (months), median (p25–p75)	2.3 (0.35–7.5)	6 (3.9–9.1)	0.065
Age <6 months, *n* (%)	8 (61.5)	12 (46.2)	0.365
Diagnosis, *n* (%)			0.858
ALL	9 (69.2)	18 (69.2)	
B or T	8–1	18–0	
AML	4 (30.8)	7 (26.9)	
MPAL	0	1 (3.8)	
CD10 (CALLA), *n* (%)			0.356
Negative	11 (84.6)	17 (65.4)	
*KMT2A*-r, *n* (%)	5 (38.4)	17 (65.4)	0.091
WBC count at diagnosis:			1
>300,000/mm^3^, *n* (%)	4 (30.8)	8 (30.8)	
Induction failure, *n* (%)			
Yes	4 (30.8)	2 (7.7)	
Status, *n* (%)			
Alive	3 (23)	17 (65.3)	
Dead before HSCT^a^, *n* (%)	7 (53.8)		
5-year OS, % (ES)	18.5 (26.3)	60.4 (28.8)	0.001
5-year OS without^a^, % (ES)	41.7 (50.4)	60.4 (28.8)	0.191

ALL, acute lymphoblastic leukemia; AML, acute myeloid leukemia; HSCT, hematopoietic stem cell transplantation; OS, overall survival; SE, standard error; WBC, white blood cell.

^a^ Death due to treatment complications or refractory disease.

For the ALL patients in the first period (1990–2005), the 5-year EFS and 5-year OS were the same at 30% (SE 14.5), and in the late period (2006–2020), the 5-year EFS and 5-year OS were 41.2 (SE 11.9) and 44.2 (SE 13.3), respectively.

For AML patients in the first period, the 5-year EFS and 5-year OS were the same at 66.7% (SE 27.2) and in the late period were 62.5 (SE 17.1) and 75 (SE 15.3), respectively. There was no statistically significant difference in EFS or OS between the early and late cohorts.

## Discussion

Although childhood leukemia is more frequently observed in male patients, infant leukemia is more common among females, a fact that is consistent in our study. Although sex does not appear to influence the outcomes in our study and other studies (6), recent results from the Children's Oncology Group (COG) describe better outcomes for girls with infant ALL (10).

The age at diagnosis was younger than 6 months in 20 (51.2%) patients, of whom 60% died. A younger age at diagnosis was associated with a poorer prognosis, which coincides with the findings of previous studies that established an age younger than 6 months as one of the main factors of poor prognosis in infant leukemia (8, 9).

Patients with infant leukemia usually have hepatosplenomegaly and extramedullary involvement. A physical examination revealed that 71% of our patients had hepatosplenomegaly and 7% presented skin infiltration, which was more frequent in patients with AML (27% vs. 15%). CNS involvement occurs more frequently in infant leukemia than in childhood leukemia; however, our findings differ from previous studies, with only 5.1% of CNS involvement at diagnosis, in contrast to results from the COG group (16.4%) (11) and the INTERFANT-06 protocol (16%) (8).

Phenotypically, infant ALL is often an early B-cell precursor and is associated with CD10 negativity and co-expression of myeloid antigens, which has been linked with these types of leukemia being resistant to standard ALL-based therapy (6, 12). Our cohort had a predominance of pro-B ALL, with 71.8% having CD10 negativity; however, this lack of CD10 antigen was not associated with worse outcomes.

The T-cell phenotype is associated with the worst outcome (13); in our cohort, only one patient had T-cell leukemia and died of progression 6 months after diagnosis.

AML accounted for 28% of the cases in this study. As in infant ALL, infant AML presents with relatively high WBC counts, hepatosplenomegaly, and CNS involvement. Unlike ALL, however, there is frequent skin infiltration (5). In our cohort, these characteristics were more frequent in ALL except for skin infiltration, which was more frequent in AML, at 27.3% (the comparison between ALL and AML is shown in Supplementary Table 2).

A total of 74% of our patients with ALL harbored *KMT2A*-r, while only 18.2% of those with AML had *KMT2A*-r. Our findings were similar to those reported in ALL, but with a lower incidence in AML, in which *KMT2A*-r is typically around 50% (8, 11, 14).

In our cohort, *KMT2*-r was not associated with poorer outcomes in either ALL or AML. The possible explanations for this are the small size of our sample; also, during the first decade of our study (1990s), cytogenetic techniques could give rise to more false negatives, as well as the fact that many of the patients without *KMT2A*-r had other factors described as poor prognosis (2), 80% of them were very young patients of age <6 months at diagnosis. In addition, this group included one patient with T-ALL and another with mixed-phenotype acute leukemia.

Treating infant ALL is challenging. Three major cooperative groups (INTERFANT, COG, and JPLSG—Japanese pediatric leukemia study group) (8, 11, 15) are conducting specific clinical trials to improve infant ALL treatment (2) and have reported several independent prognostic factors for infant ALL, including *KMT2A*-r, age, WBC count at diagnosis, and a poor prednisone response on day 8 (2).

In our patients with ALL, induction failure and relapse occurred in 15% and 36%, respectively. The median duration of first remission before relapse was 4 months. These results are consistent with larger studies that suggest that almost half of all infants with ALL will relapse, with most relapses occurring early (median of 10 months) (8, 9).

The 5-year OS in our ALL patients was 38.4% (SE 10), an abysmal result compared with the current outcomes reporting for IL. These results have been different over time, with 30% OS in the first period of the cohort (ES 14.5), when patients were treated in pediatric ALL protocols that are not specific for infants, and in general, there were fewer therapeutic options to improve the results (Table 2). In the second period, the results have improved, obtaining an OS of 44.2% (SE 13.3), a result similar to what is currently reported (8).

Outcomes for infants with AML are comparable with those of older children with similar cytogenetic features. Given the similar prognosis and therapeutic response, infants are generally treated with the same clinical trial protocols as older children (16). Induction failure and relapse occurred in 27% and 18% of our cohort with AML, respectively, and the median duration of the first remission before relapse was 10 months. In the early period of our cohort where the 5-year OS was 66.7% (ES 27.2) and in the late period 75% (ES 15.3), we see considerably better results than in ALL, as what has been reported in other series, and also an improvement in the most recent results.

The role of HSCT in infant leukemia is controversial. Regardless of diagnosis, HSCT in this vulnerable age group can prove difficult (16). For AML, HSCT indications are similar to those for older patients and are based on molecular features and therapeutic responses. A consolidative HSCT plays an important role for pediatric patients with high-risk AML, and this potential transplant benefit is extrapolated to infants, although large series of infants have not been examined (5).

The role of HSCT in infant ALL is less clear. An HSCT indication during the first complete remission has not yet been uniformly established for this age group, with the most high-risk infants in European and Japanese (but not US) protocols being stratified to HSCT according to the age at diagnosis, WBC counts above 300 × 10^9^/L, and a poor prednisone response (17).

**Figure 1:**
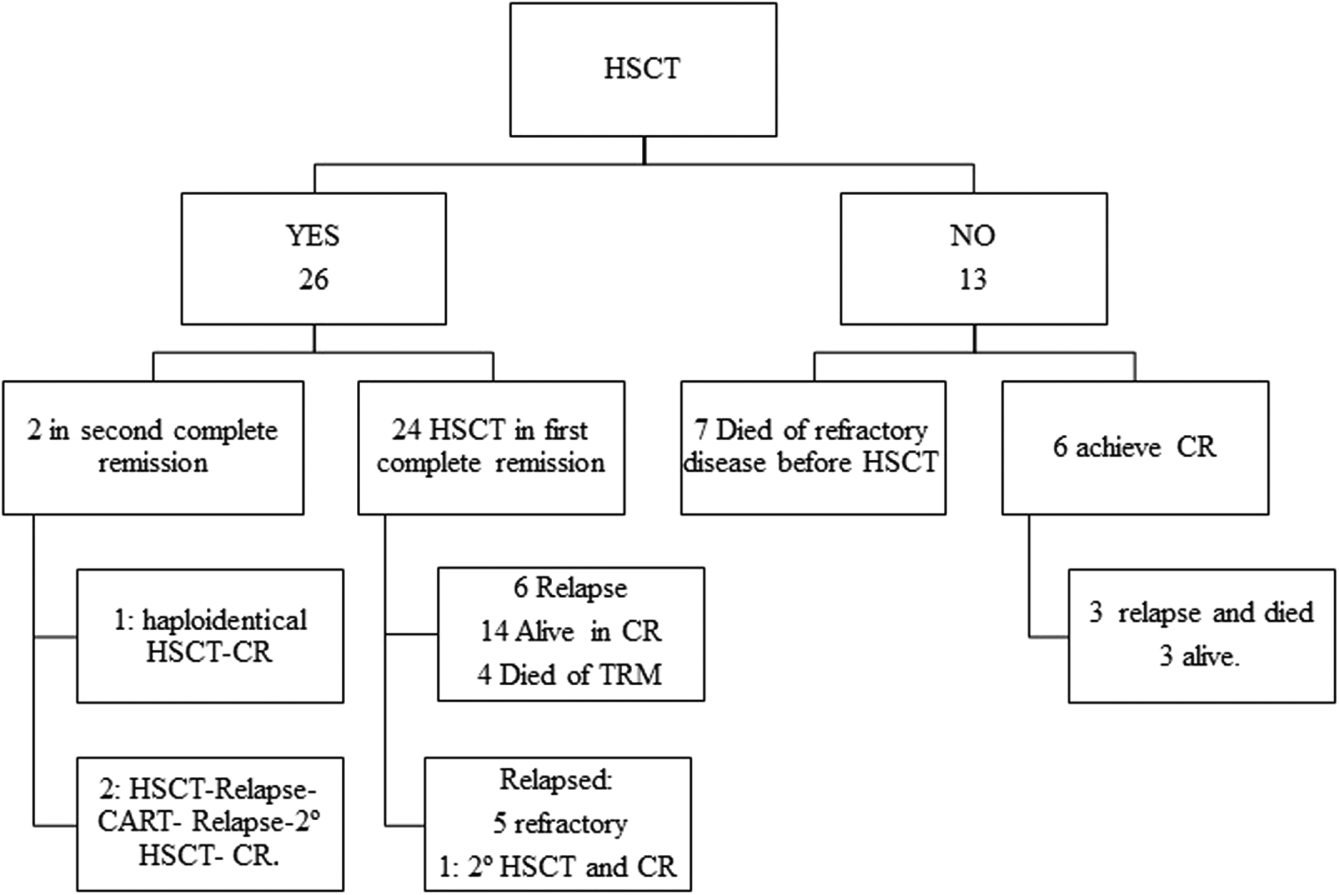
Follow-up of transplanted patients. CR, complete remission; HSCT, Hematopoietic stem cell transplantation; TRM, transplant-related mortality.

In our cohort, 26 (66.6%) patients underwent HSCT, of which 24 (92.3%) during their first complete remission (Figure 1). We saw an evolution from the first period when, according to local protocols, some patients underwent autologous HSCT as a consolidation treatment, a practice that has decreased in the most recent period (Table 2). On the other hand, we see an increase in the use of haploidentical HSCT, according to what has been reported in our country with the acquisition of experience and graft manipulation techniques in the recent decades (18). The 5-year OS for the patients who underwent HSCT was 60.4% vs. 18.5% for the non-transplanted patients, similar to that reported in INTERFANT-99 (a 4-year disease-free survival of 50% for HSCT vs. 37% for chemotherapy alone) (9). However, group comparisons that excluded the patients who were unable to undergo transplantation due to refractoriness/relapse or death during treatment showed no statistically significant differences between the HSCT and the non-HSCT groups (Table 4). TRM was 31.6%, consistent with the substantially higher risk of TRM reported for infants compared with older children (17).

It is important to note that this study included various conditioning regimens and a variety of donor sources, which makes it difficult to evaluate the true benefit of a particular transplantation approach. HSCT is currently recommended for high-risk patients, although the occurrence of relapse after HSCT is also common (15, 19). In our study, 17.9% of the patients relapsed after HSCT.

Advances in molecularly targeted therapies and immunotherapy present promising opportunities for potential improvements. The incorporation of novel treatments in this young population suggests the possibility of reducing the toxicity and late effects associated with classical cytotoxic chemotherapy and HSCT (16).

Chimeric antigen receptor (CAR) T-cell therapies have shown exciting promise for pediatric patients with relapsed/refractory leukemia. Although antigen targets for ALL (CD19, CD22) and AML (CD33, CD123) could be useful for treating infant leukemia (20, 21), there are limitations to their use, including the development of lineage switching as an escape mechanism, especially in cells with *KMT2A*-r, in addition to the difficulty of apheresis and the subsequent manufacturing of CAR-T cells in infants (22, 23). In our cohort, one patient underwent CAR-T-cell therapy in 2020. This patient received CAR-T cells after a post-HSCT relapse; however, 9 months after administering the CAR-T cells, the patient experienced an isolated extramedullary relapse and underwent a second HSCT using a haploidentical donor, after which the patient remained in complete remission (24).

With a median follow-up of 15 months, the 5-year EFS and 5-year OS were 43.6% and 46.5%, respectively, similar to the results published in other series of infant leukemia (5, 25, 26). The closeness between EFS and OS reflects the difficulty of rescuing patients who relapse.

**Figure 2:**
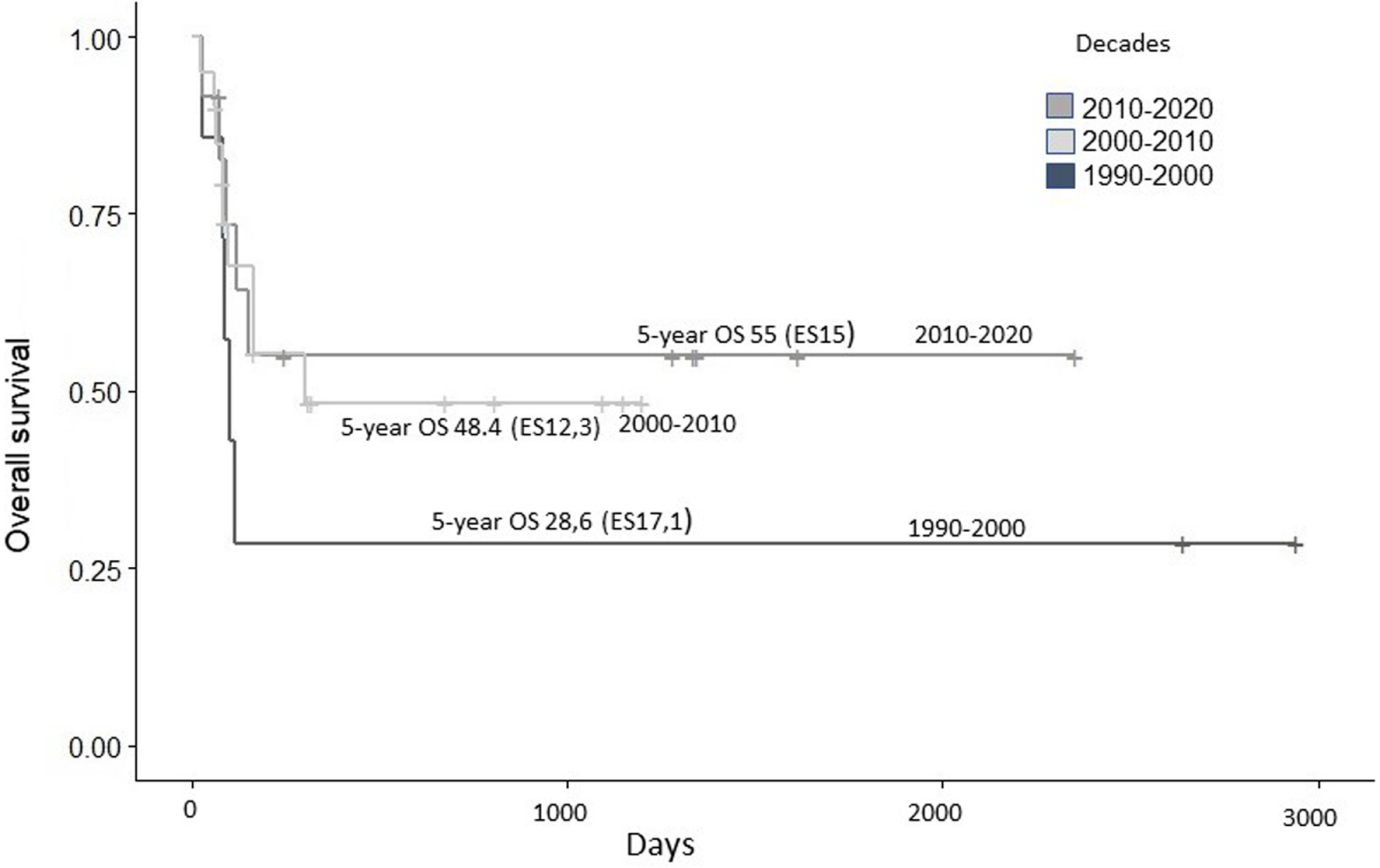
Overall survival between earlier versus later period of time.

We can see the improvement in the results in our cohort from the 1990s to the present (Figure 2). This is a reflection of the implementation of new therapeutic strategies, the advance in the knowledge of the biology of IL and, in turn, the gain in expertise in the treatment of such vulnerable patients.

Our results suggest that HSCT is a good and efficient option for treating selected patients. However, there remains a major issue in deciding which patients should undergo transplantation. More studies with larger patient groups are needed to re-evaluate the eligibility criteria for HSCT in this patient group.

The main risk factors that affected survival in our study were an age younger than 6 months and a poor response to induction therapy. There were no differences in the patients with *KMT2A*-r compared with the results reported by other studies.

Sensitivity to the initial therapy and good response at the end of the induction therapy were the determining factors that improved survival in our cohort, which are independent of other factors.

It is important to continue stratifying the treatment according to the factors that confer a poorer prognosis so as to improve the results for infant patients, whose condition does not behave like childhood leukemia and therefore does not respond to the same treatments.

## Data Availability

The original contributions presented in the study are included in the article/Supplementary Material, further inquiries can be directed to the corresponding author.
