# Supplementary material for: Younger age and induction failure predict outcomes in infant leukemia: 30 years of experience in a tertiary center
Source: Front Pediatr. 2023 May 30;11:1166176. doi: 10.3389/fped.2023.1166176 (PMC10263122; doi:10.3389/fped.2023.1166176)
Supplement: Supplementary file 1 [file Table1.docx]

Supplementary Material

**Title: Younger age and induction failure predict outcomes in infant leukemia: 30 years of experience in a tertiary centre**

TABLE 1: Hematopoietic stem cell transplantation type and conditioning

|  | HSCT Type | Diagnosis | MLL-r | Sex | Age (months) | Conditioning | ProphylaxisGvHD | Status |
| --- | --- | --- | --- | --- | --- | --- | --- | --- |
| #1 | Autologous | ALL-B | No | Female | 9 | Busulfan, CY | No | Alive |
| #2 | Autologous | ALL-B | No | Female | 8 | Busulfan, Etoposide | No | Alive |
| #3 | Autologous | ALL-B | Yes | Female | 7 | Busulfan, etoposide, CY | No | Alive |
| #4 | Autologous | AML | No | Male | 7 | Busulfan, etoposide, CY | No | Alive |
| #5 | MUD | ALL-B | Yes | Female | 10 | Busulfan, CY, ATG | CsA, MTX | Alive |
| #6 | MUD | AML | No | Female | 16 | Carmustine, cytarabine, amsacrine, ATG | CsA | Dead |
| #7 | MRD | AML | No | Male | 17 | Busulfan, etoposide, CY | CsA | Alive |
| #8 | MUD | AML | No | Female | 17 | Busulfan, etoposide, CY, ATG | CsA | Alive |
| #9 | MUD | ALL-B | Yes | Female | 15 | Busulfan, etoposide, CY, ATG | CsA | Alive |
| #10 | MRD | ALL-B | Yes | Male | 9 | Busulfan, etoposide, CY | CsA | Alive |
| #11 | MUD | ALL-B | No | Female | 18 | Busulfan, fludarabine, ATG, thiotepa | CsA, MTX | Alive |
| #12 | MUD | ALL-B | Yes | Female | 8 | Busulfan, CY, thiotepa, ATG | CsA, MTX | Dead |
| #13 | MUD | ALL-B | Yes | Male | 10 | Busulfan, fludarabine, ATG, thiotepa | CsA | Dead |
| #14 | MUD | ALL-B | Yes | Female | 15 | Busulfan, fludarabine, ATG, thiotepa | CsA+MTX | Alive |
| #15 | MUD | ALL-B | No | Female | 6 | Busulfan, fludarabine, ATG, thiotepa | CsA+MTX | Dead |
| #16 | MUD | ALL-B | Yes | Female | 4 | Busulfan, fludarabine, ATG, thiotepa | CsA+MTX | Dead |
| #17 | MUD | ALL-B | Yes | Female | 13 | Melphalan, fludarabine, thiotepa, TLI | MMF | Alive |
| #18 | Haploidentical | ALL-B | Yes | Female | 6 | Busulfan, melphalan, ATG | CsA | Dead |
| #19 | Haploidentical | ALL-B | Yes | Male | 6 | Busulfan, etoposide, CY, ATG | CsA | Dead |
| #20 | Haploidentical | ALL-B | Yes | Female | 13 | Busulfan, fludarabine, ATG, thiotepa | CsA | Dead |
| #21 | Haploidentical | ALL-B | Yes | Male | 4 | Busulfan, fludarabine, ATG, thiotepa | CsA | Alive |
| #22 | Haploidentical | ALL-B | Yes | Male | 6 | Busulfan, fludarabine, ATG, thiotepa | CsA | Alive |
| #23 | Haploidentical | AML | Yes | Female | 11 | Melphalan, fludarabine, ATG, thiotepa | MMF | Alive |
| #24 | Haploidentical | MPAL | No | Female | 13 | Melphalan, fludarabine, thiotepa, TLI | MMF | Dead |
| #25 | Haploidentical | AML | Yes | Female | 10 | Melphalan, fludarabine, thiotepa, TLI | CsA | Alive |
| #26 | Haploidentical | ALL-B | No | Female | 18 | Melphalan, fludarabine, thiotepa, TLI | No | Alive |

ALL, acute lymphoblastic leukemia; AML, acute myeloblastic leukemia; ATG, antithymocyte globulin; CsA, cyclosporine; GvHD,graft versus host disease; HSCT, hematopoietic stem cell transplantation; MRD, matched related donor; MUD, matched unrelated donor; MPAL, mixed phenotype acute leukaemia; MLLr, MLL gen rearrangements; CY, cyclophosphamide; TLI, total lymphoid irradiation

TABLE 2: Comparison between acute lymphoblastic leukemia and acute myeloid leukemia

| INFANT LEUKEMIA | ALL | AML |
| --- | --- | --- |
| Patients, n (%) | 27 (69) | 11 (28) |
| Sex, n (%)  Boys  Girls | 10 (37)  17 (63) | 5 (45.5)  6 (54.5) |
| Age (months), mean (SD) | 5.38 (3.3) | 5.87 (4.3) |
| CD10 (CALLA), n (%)  Negative | 16 (59) | 11 (100) |
| KMT2A-r, n (%) | 20 (74) | 2 (18) |
| WBC at diagnosis: cells/mm^3^  Median (p25-p75) | 152.140  (41.400-470.000) | 17.830  (8.600-80.750) |
| WBC >300.000/mm^3^, n (%) | 10 (37) | 1 (9) |
| Skin infiltration, n (%) | 4 (15) | 3 (27) |
| CNS involvement, n (%) | 2 (7) | 0 |
| Induction failure, n (%) | 3 (11) | 3 (27) |
| Relapse, n (%) | 11 (41) | 2 (18) |
| HSCT, n (%) | 18 (67) | 7 (64) |
| Status, n (%)  Alive | 12 (44.4) | 8 (72.7) |

ALL, acute lymphoblastic leukemia; AML, acute myeloid leukemia; CNS, central nervous system; HSCT, hematopoietic stem cell transplantation; IQR, interquartile range; OS, overall survival; WBC, white blood count.
